# Retraction: LncRNA AK045171 protects the heart from cardiac hypertrophy by regulating the SP1/MG53 signalling pathway

**DOI:** 10.18632/aging.204530

**Published:** 2023-02-15

**Authors:** Li Xu, Hongjiang Wang, Feng Jiang, Hao Sun, Dapeng Zhang

**Affiliations:** 1 Heart Center and Beijing Key Laboratory of Hypertension, Beijing Chaoyang Hospital, Capital Medical University, Beijing 100020, China

**Keywords:** LncRNA, cardiac hypertrophy, SP1/MG53

**This article has been retracted:** Aging has completed its investigation of this paper. The investigation found that the text and figures overlap with an article by different authors published in another journal [1], which has already been retracted. The **Figure 1C** “sham” image is identical with “TAC” image in **Figure 2C** from the Aging paper and the **Figure 2E** “TAC” image from the aforementioned other paper [1]. Two additional panels of **Figure 2C** also are identical to two panels of **Figure 2E** in the other paper [1]. **Figure 2B** - H&E staining of the heart tissue - bottom panels “TAC” and “TAC+Ad-AK045171” are identical with the bottom “TAC” and “sham” images of **Figure 4F** of the other paper [1]. The authors informed the journal that they have agreed with the retraction.

The Administration of the Beijing Chaoyang Hospital, Capital Medical University was notified about the retraction by Aging Journal.
